# Removal Bridge or Plate Work

**Published:** 1893-07

**Authors:** Chas. L. Van Fossen

**Affiliations:** Kansas City, Kan.


ARTICLE II.

### REMOVAL BRIDGE OR PLATE WORK.

DR. CHAS. L. VAN FOSSEN, KANSAS CITY, KAN.

The first consideration brought to our minds when a bridge is spoken of is the anchors. I want my position understood when I say that good solid teeth or roots for anchorage of removable bridges should invariably be used. By this premise I would indicate that I consider it even more necessary than in stationary work; because the insertion and withdrawal of the piece will of necessity cause strain on the anchors.

I speak of the anchors as of prime importance, for I see and hear of so many pieces, carefully designed and constructed, yet in a year's time we see the grand monument of esthetic bridge-work fallen.

Every failure of bridge-work carries some one over to that army of opponents to all classes of bridge-work. There has been so much work attempted in replacement of teeth by anchorage to natural teeth or roots, so positively against all sense of reason, all laws of science or mechanics, that it is not to be wondered that we are constantly meeting people who are most pronounced in their opposition to all classes of bridge work, and who seem to think you are doing them a wrong to even intimate to them that such work can, or is, being done. They may have good cause for feeling as they do; possibly they, or some of their friends, have been the victims of misapplied knowledge; still the fact of your seeing or hearing more cases as failures, gives us no criterion to stamp the whole line of work a failure.

There seems to be no other field so open to practical genius as is found in dental prosthesis. There are myriads of ideas, ensembled and grouped together, of part development, and yet it does seem as if we were but on the dawning of the day, as the sun barely peeps over the horizon, as to what might be done in the future.

The first thing we consider, after the tooth or root anchors are decided on, is the root protection. This is made by trimming the sides of each anchor to a perpendicular position by means of corundum, emery, and sand-paper discs, and banding each anchor accurately. To simplify I will describe the anterior and posterior anchors separately.

In the anterior teeth we utilize only the root, trimming down in the ordinary manner for setting a Richmond crown, with one exception, and this is the advisability of slanting the root from the labial to the lingual surface, to allow of as high a ledge in the rear as circumstances will permit, trimming the band on the labial surface to almost the gum margin. Next we solder a flat cap over the band and trim down the edges accurately. We next enlarge our pulp canal as large as possible to still retain sufficient strength, and turn our attention to the pin, which is made of either gold clasp metal or platinum and gold. Taking a heavy wire, bend about double, and inverting the loop end down, add twenty or twenty-two k. gold to the loop by melting till we have quite a ball or knob as large as the top of our root will admit, trimming this pin up to loosely fit the root. We next make a tube of No. 30 platinum to fit the pin accurately, soldering the tube with pure gold. Puncturing the cap of one root protection, we drive the pin and pin covering through as far into the root as possible. We will have a little surplus of the platinum sheath or tube, which we split in several places and burnish down to the cap. Next carefully removing the pin to not displace the tube, we remove the root protection with the tube, and invest upside down, being careful to fill the tube with the investment, and thereby keep the solder out, heating up the investment solder altogether. We now proceed to make a Richmond crown for the platinum covering of the root, being careful not to let the band impinge on the gum, as its removal and subsequent replacement a number of times daily would start up a serious gum irritation. We do not consider the pin at all in making the crown, but make it pinless, and when the whole piece is complete trim the top of the pin to a round centre, and thread in your screw plate, and place in the root tube, drop a piece of wax in the centre of the crown, press to place. This will mark the position of the pin and allow you to take a drill the size of the screw, and drill a hole in the crown, and then thread the hole the same as the pin. We can now screw our pin in and rivet at the top, and any subsequent repairs made with very little inconvenience.

In the posterior attachment we allow the inner band of platinum to extend nearly as high as the natural tooth was, leaving only sufficient space between the band and articulating teeth to allow of a quite heavy solid gold cap, capping the band in the usual manner, *flat*. Make the pin as heavy as possible and cover with the tube as described for the anterior crowns.

With the exception that we make the ball at the top much heavier and the pins separated much more, and make a partition wall in our tube to fit between the two prongs of the pin, proceed to connect the tube and root covering as described, and, after polishing, set with cement to place.

I neglected to state that this tooth covering should always be made some smaller at the top than at the gum margin.

We next make a telescope crown of twenty or twenty-two k. gold to fit this inner platinum sheaf, trimming off flat at the top, and next mold a solid gold cap to make a thorough articulation with the corresponding teeth.

A very nice method of obtaining a perfect articulation being to pound a bullet into your die-plate on the tooth you desire, file flat and lay on top the cap, and allow the patient to close the jaws firmly. The opposite teeth will press into the lead easily, and allow you to trim down with a knife.

When perfect, drop a little sticky wax on a round stick and place the lead on it, and proceed to mold your cap in the cuttle-fish bone as usual. By this method you have no grinding of the cap after finished.

Soldering the cap now to the telescope band, we have our anchor complete, with the exception of the pin in the cap, which we screw and rivet to place.

We are now ready to consider the attachment of the intervening or bridge teeth. Here we are allowed a variety of methods. The only stipulation I would make is that the gum should be saddled to allow it to assist in standing the strain.

We can swage a rim of gold to fit the gum quickly by taking a plaster impression and drying out; flow in fusible metal and proceed to burnish the rim to place, which can readily be done by annealing a few times. Soldering the saddle to the two anchors, and a few cleates on the saddle, proceed to vulcanize the teeth to the saddle in the ordinary manner of attaching to a gold plate.

Again, in the lower jaw, we may make a very inexpensive case by attaching the dummies together with Watts metal, and soft solder this to the two anchors.

Again, by simply soldering a strong bar of gold or platinum between the two anchors, vulcanizing the teeth between.

If you want more elaborate and extensive work, swage your rim, back your teeth and solder to the saddle, rim the labial surface, and you have a whole gold case.

The advantages I claim for this class of work over stationary bridge-work are :

1st. Absolute cleanliness, as the patient can remove and cleanse at will.

2nd. A more natural appearance of the work, as by the use of rubber or celluloid for attachment we can use teeth without the heavy gold coverings, as a breakage does not necessitate much work in repairing.

3rd. More extensive work, as the teeth used as anchors do not have to stand the blunt of the forces of mastication.

4th. Extension bridges, as the gum will stand the strain.

5th. Allowing us to more easily reproduce nature on utilizing gum-blocks where, by the ravages of disease or marked shrinkage of the alveola, such are indicated.

6th. A more natural feeling in the mouth, as no spaces need be left to insure cleanliness.—*Items of Interest.*

